# Identification of Phenotypic Diversity and DArTseq Loci Associated with Vitamin A Contents in Turkish Common Bean Germplasm Through GWAS

**DOI:** 10.3390/plants14050776

**Published:** 2025-03-03

**Authors:** Yeter Çilesiz, Muhammad Tanveer Altaf, Muhammad Azhar Nadeem, Amjad Ali, Uğur Sesiz, Ahmad Alsaleh, Ahmet İlçim, Mehmet Sertaç Özer, Tunahan Erdem, Israr Aziz, Sheikh Mansoor, Tolga Karaköy, Faheem Shehzad Baloch

**Affiliations:** 1 Department of Field Crops, Faculty of Agricultural Sciences and Technologies, Sivas University of Science and Technology, Sivas 58140, Türkiye; yetercilesiz_mbg@hotmail.com; 2 Department of Field Crops, Faculty of Agriculture, Recep Tayyip Erdoğan University, Rize 53300, Türkiye; 3 Department of Plant Protection, Faculty of Agricultural Sciences and Technologies, Sivas University of Science and Technology, Sivas 58140, Türkiye; amjadbzu11@gmail.com (A.A.); tolgakarakoy73@hotmail.com (T.K.); 4 Department of Field Crops, Faculty of Agriculture, Sirnak University, Sirnak 73000, Türkiye; usesiz@sirnak.edu.tr; 5 Molecular Genetic Laboratory, Institute for Hemp Research, Yozgat Bozok University, Yozgat 66200, Türkiye; aal_saleh@yahoo.com; 6 Science and Letter Faculty, Biology Department, Hatay Mustafa Kemal University, Hatay 23323, Türkiye; ailcim@mku.edu.tr; 7 Department of Food Engineering, Faculty of Engineering, Cukurova University, Adana 01330, Türkiye; msozer@cu.edu.tr; 8 Department of Agricultural Machinery and Technologies Engineering, Cukurova University, Adana 01330, Türkiye; terdem@cu.edu.tr; 9 Interdisciplinary Graduate Program in Advanced Convergence Technology and Science, Jeju National University, 102 Jejudaehak-ro, Jeju 63243, Republic of Korea; israraziz62@gmail.com; 10 Department of Plant Resources and Environment, Jeju National University, Jeju 63243, Republic of Korea; mansoorshafi21@gmail.com (S.M.); balochfaheem13@gmail.com (F.S.B.); 11 Department of Biotechnology, Faculty of Science, Mersin University, Mersin 33343, Türkiye

**Keywords:** *Phaseolus vulgaris* L., biofortification, hidden hunger, germplasm characterization, GWAS

## Abstract

Biofortification has emerged as an important approach for improving minerals and vitamin deficiencies through the application of agronomic and biotechnological methodologies. Vitamin A, one of the most deficient vitamins, disproportionately affects children in developing countries, highlighting the urgent need for vitamin A-enriched cultivars. The present study aimed to characterize common bean germplasm for vitamin A contents and to identify the genomic regions associated with this trait. A total of 177 common bean landraces and 6 commercial cultivars were evaluated under five environments and two locations. Analysis of variance revealed highly significant environmental effects and genotype × environment interactions. Across all years and all locations, Bilecik-6 exhibited the lowest vitamin A contents (1.67 µg/100 g dry seed), while Civril-Bolu had the highest (3.23 µg/100 g dry seed). Landraces from Balıkesir province were found to be rich in vitamin A content and should be considered as potential genetic resources for common bean biofortification. Additionally, a genomic region located on Pv06 was identified as being linked to vitamin A content. The genomic and genetic resources identified in this study will be valuable for the breeding community aiming to develop vitamin A-enriched common bean cultivars.

## 1. Introduction

Global agriculture is increasingly threatened by the dual challenges of a rapidly growing population and accelerating climate change. Climate change poses a significant threat to our agriculture by substantially reducing yields through various biotic and abiotic stresses [1]. Climate change scenarios are expected to become more adverse in the upcoming years [2]. Therefore, this situation highlights the urgent need to develop climate-resilient cultivars to ensure agriculture sustainability. Besides climate change, the global population is expected to surpass 9 billion by 2050, requiring a 70% increase in agricultural production to meet food demand [3]. Food scarcity remain a major global issue, exacerbated by factors such as economic instability, armed conflicts, and climate change. By the end of 2022, an estimated 222 million people experienced severe food insecurity [4,5]. The number of people who do not have enough food increased significantly, reaching 720–811 million between 2020 and 2021 [6]. Therefore, it is very important to make a serious effort to increase crop yield. Besides crop yield, quality is also becoming a key issue concerning human health. Most of our routine diet fails to provide sufficient mineral elements required for the growth and development of our body. The existence of several micronutrient deficiencies despite an adequate energy intake is frequently referred to as ‘hidden hunger’ or malnutrition [7]. Hidden hunger affects an estimated two billion individuals [8,9], most of whom suffer from anemia, which affects one-fourth of the world’s population [10]. The most often limiting micronutrients in the diet are iron, zinc, iodine, and vitamins (especially vitamins A and B9) which often result from an energy-dense but nutrient-poor diet.

Vitamins are the organic compounds that support normal metabolic processes while being present in small amounts in food. Their deficiency can cause health problems, and their replenishment helps alleviate deficiency symptoms [11]. As micronutrients, vitamins are not naturally synthesized by the human body [12], and must be obtained from the diet [13]. They are unique and very important in terms of being low in concentration, allowing the molecular events associated with metabolism to occur [14]. Currently, 13 vitamin compounds are classified into two categories based on their solubility: fat-soluble vitamins (vitamins A, D, E, and K) and water-soluble vitamins (the B-complex vitamins (vitamins B1, B2, B3, B5, B6, B8, B9, and B12) and vitamin C) [15]. Beans are a highly nutritious food and a rich source of vitamins. They are especially rich in B vitamins. Beans contain beta-carotene, which is provitamin A. This is converted into vitamin A in the body, and is beneficial for vision, skin health, and immune function. Some types of beans, especially green beans, contain vitamin K. Vitamin K regulates blood clotting and supports bone health. Since beans are generally low in vitamin C, it may be beneficial to supplement them with other foods (e.g., fruits) to meet your vitamin C needs [16].

Among the fat-soluble vitamins, vitamin A is colorless and exists in the form of a long-chain unsaturated alcohol with five double bonds in its structure. The richest sources of vitamin A are fish oil, egg yolk, milk, liver, and carrots. Vitamin A is one of the micronutrients that play a key role in various biological activities such as embryo development, bone and organ formation, the function of the sense of sight, the formation of the immune system, and reproduction in the human body [17]. Vitamin A has important functions in the body, and its deficiency can lead to various diseases, including night blindness, dry eyes, growth retardation in children, a weakened immune system, and dry skin [18]. It is recognized as one of the least abundant vitamins in the body, particularly in children from developing countries. It is estimated that 30% of children under the age of five suffer from vitamin A deficiency worldwide, and approximately 2% of deaths at this age are attributed to this deficiency [19]. One of the leading causes of child blindness is vitamin A deficiency. Moreover, vitamin A deficiency can be life-threatening for pregnant or breastfeeding women [20]. A failure to consume adequate amounts of vitamins in daily life results in micronutrient deficiency, also known as ‘hidden hunger’. This deficiency poses a significant global health threat, affecting more than two billion people [21].

Legumes are an important pillar of our agricultural production system, and their benefits for sustainable agriculture are universally known. Common bean is one of the most important legume crops, serving as a source of daily nutrition to a large number of populations all over the world [22]. Mesoamerica is considered to be the origin of the common bean, and its domestication resulted in the formation of two unique gene pools, i.e., the Mesoamerican and Andean gene pools [23]. The common bean is a rich source of protein, carbohydrates, essential minerals, and vitamins required for normal growth. These nutritionally rich qualities make a common ‘grain of hope’ [24]. Today, the common bean is grown globally. In 2019, about 27 million tons of green beans and 29 million tons of dry beans were produced globally, a total common bean production that has grown 36% in the last 10 years (FAO, 2019). Global common bean production has accounted for 27,545,942 tons by 2020 [25]. Türkiye, which imports common beans from Europe, houses a large number of common bean germplasm that are genetically closer to their wild forms, as compared to other European varieties. Türkiye is recognized as the third-leading producer of common beans worldwide, with an annual production quantity of 212,758 tons in 2019, followed by the Mediterranean region. Common beans have since become part of Turkish dishes, either as “fresh pods” or “dried seeds” [25].

Germplasm characterization is considered a prerequisite for breeding activities, as it facilitates researchers to identify novel variations that can be helpful for the breeding community in developing new cultivars [25]. Genetic variation can be investigated at both phenotypic and genotypic levels. Very few studies have been carried out to screen common bean germplasm and its vitamin A contents. Köse et al. [26] investigated the vitamin content of two bean genotypes (*Phaseolus vulgaris* L. cvs. Yenice and Pinarli) commonly grown in the Çamoluk region of Turkey. They found that the retinol (vitamin A) content in the Pinarli genotype was 4.15 µg 100 g^−1^, which was significantly higher than in the Yenice genotype. Genomic studies seeking to identify genetic variants often use either QTL mapping or GWAS. However, QTL mapping is limited by a few recombinations, which are laborious, time-consuming processes, and it produces population-specific QTLs [27]. These constraints have led the scientific community to consider GWAS as a complementary approach for studying genomic regions associated with traits of interest [28]. Bulk segregant analysis (BSA) and conventional QTL analysis are QTL mapping techniques used in combination with whole-genome resequencing (WGS) to map genes of interest in crops such as soybean, melon, and rice [29]. BSA is a technique used to group genetic diversity based on specific phenotypic differences. This technique, which is typically used to understand differences between two extreme phenotypes, does not require large populations and provides fast results. Conventional QTL analysis, on the other hand, is a more detailed approach that aims to identify specific loci on the genetic map. While DArTseq is generally used to rapidly detect genetic diversity, BSAseq is used to understand genetic interactions and the underlying basis of specific phenotypic traits [30].

To date, there is no available report claiming the identification of marker-–trait association for vitamin A contents in common bean germplasm. The present study aimed to investigate vitamin A diversity in Turkish common bean germplasm and to identify marker–trait associations for vitamin A contents.

## 2. Results

Analysis of variance (ANOVA) was performed to determine the effects of genotype, genotype × environment, and their interaction (G × E) on vitamin A content in five different environments and two different locations. As a result of the analysis, it was determined that the interaction between environment and genotype × environment was statistically highly significant in terms of vitamin A content, while genotypic effects (*p* < 0.01) were not significant (Table 1).

**Figure 1:**
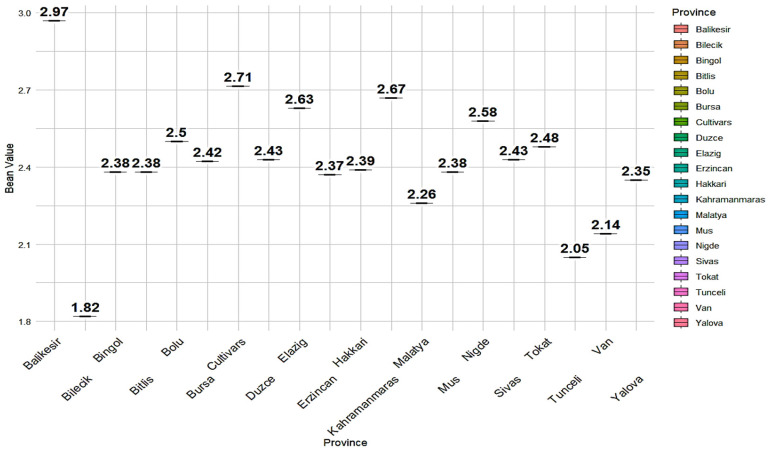
Province-based vitamin diversity in Turkish common bean germplasm.

Vitamin content profiling was performed, and this exhibited the diversity of vitamin A content across the studied germplasm under the environmental conditions examined (Supplementary Table S2). The minimum, maximum, and mean vitamin A contents during five environments and two locations are presented in Table 2. At the Bolu location (2017 and 2018), the Civril-Bolu landrace exhibited the highest vitamin A content, i.e., 3.22 µg/100 g dry seed, 3.35 µg/100 g dry seed, respectively. Bilecik-6 and Sivas-62 landraces reflected minimum vitamin A contents for 2017 and 2018, respectively. At the Sivas location, Balıkesir-18, Balıkesir-17, and Civril-Bolu landrace reflected maximum vitamin A contents, i.e., 3.26, 3.24, and 3.23 µg/100 g dry seed, respectively. When the data of all locations was combined, Civril-Bolu (3.23 µg/100 g dry seed) and Bilecik-6 (1.67 µg/100 g dry seed) landraces reflected maximum and minimum vitamin A contents. Province-based vitamin A contents was also investigated, revealing that accessions from Balıkesir were rich in vitamin A, whereas accessions from the Bilecik province had the lowest vitamin A content (Figure 1).

**Figure 2:**
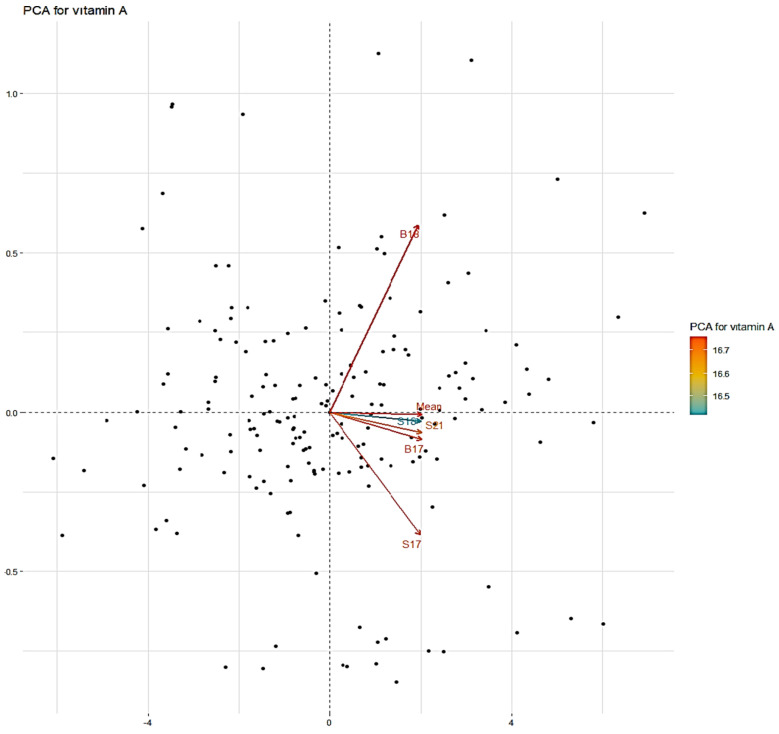
PCA for vitamin A contents in Turkish common bean germplasm.

**Figure 3:**
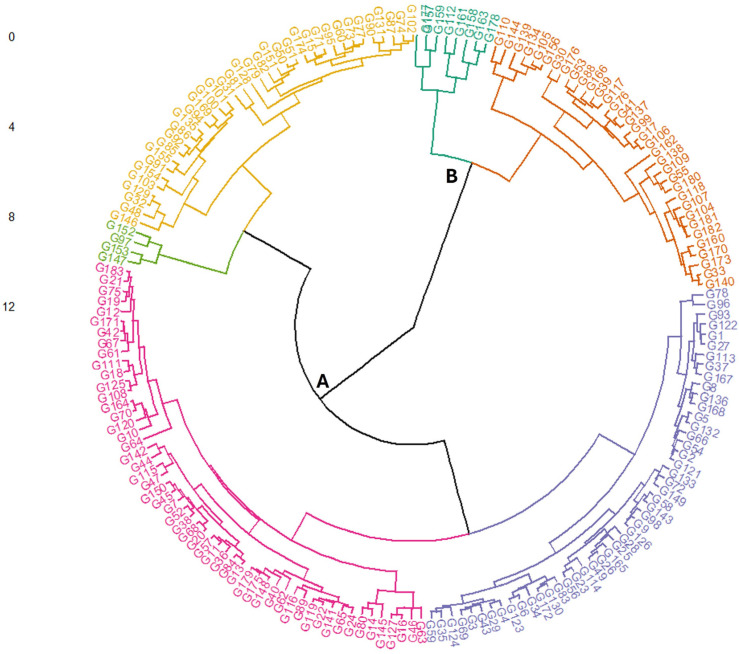
Hierarchical clustering dendrogram based on vitamin A content in Turkish common bean germplasm. The dendrogram illustrates the genetic diversity among accessions, categorizing them into distinct clusters based on their vitamin A concentrations. Each genotype is assigned to a unique identifier (G1 to G183), corresponding to the accession codes used in this study. These codes are represented in Supplementary Table S1.

**Figure 4:**
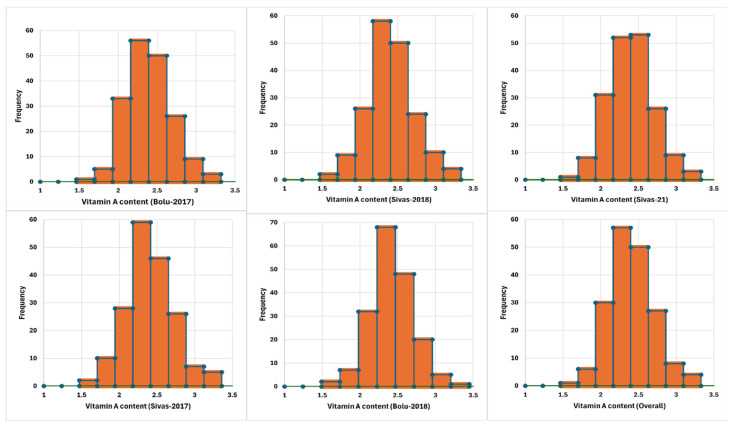
Vitamin A content distribution in Turkish common bean germplasm.

Principal component analysis (PCA) was performed, and five main components constituted 100.00% of the total variation (Figure 2 and Table 3). The PCA1 axis accounted for 98.419% of the total variation, with the highest positive eigenvalue observed in the data obtained from the Sivas location in 2018. The PCA2 axis explained 0.668% of the variation. On the other hand, the PCA3 axis accounted for 0.429% of the variation, with the data from the Sivas location in 2021 being the most characteristic feature of this factor. The PCA4 and PCA5 axes explained 0.323% and 0.160% of the total variation, respectively. A dendrogram was constructed based on the vitamin A content, dividing the studied germplasm into two distinct populations: A and B. Population A was larger and showed greater genetic diversity than Population B (Figure 3). A frequency distribution analysis was conducted to verify the data distribution, revealing a normal distribution of vitamin A across all conditions (Figure 4). Once the stability analysis was completed, ten of the most stable accessions were identified and examined (Table 4).

Marker–Trait Association for Vitamin A Contents.

**Figure 5:**
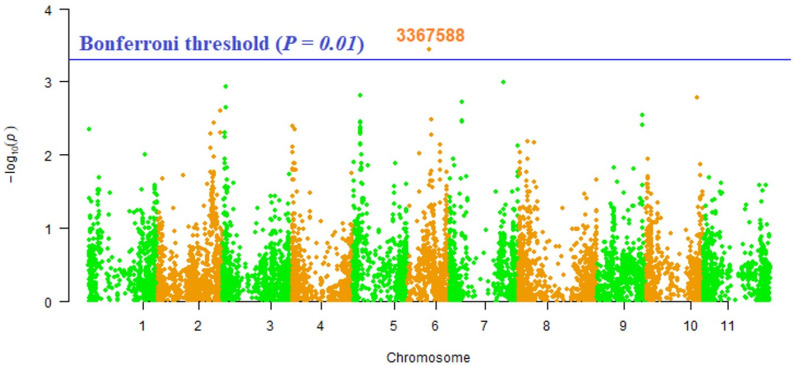
Manhattan plot of vitamin A in common beans using DArTseq markers.

A total of 7900 DArTseq markers were used for marker–trait association. Among these markers, only one marker (3367588) distributed on Pv06 showed a significant association with vitamin A (Table 5; Figure 5).

## 3. Discussion

Analysis of variance (ANOVA) was performed to determine the effects of genotype, genotype × environment, and the interaction of both (G × E) on vitamin A content in five different environments and two different locations. As a result of the analysis, it was determined that the interaction between environment and genotype x environment was statistically highly significant in terms of vitamin A content, while genotypic effects (*p* < 0.01) were not significant (Table 1). There is no available report sharing information regarding the effect of G × E interaction on vitamin A contents in common beans. However, Mengesha et al. [31] aimed to explore the G × E interaction effect on vitamin A contents in maize and identified similar results to our study.

A good range of variations was observed for vitamin A contents in Turkish common bean germplasm. During both years 2017 and 2018 at the Bolu location and 2021 at the Sivas location, Civril-Bolu landrace showed the highest vitamin A content, i.e., 3.22 µg/100 g dry seed, 3.35 µg/100 g dry seed, and 3.23 µg/100 g dry seed, respectively. Balıkesir-18 landrace showed the highest vitamin A content with 3.26 µg/100 g dry seed in 2017 in the Sivas location and the Balıkesir-6 genotype, with 3.24 µg/100 g in 2018 at the same location. Considering the average of all years and all locations in terms of vitamin A contents in Turkish common bean landraces, the Bilecik-8 landrace showed the minimum vitamin A contents (1.67 µg/100 g dry seed), and the Civril-Bolu landrace reflected the highest vitamin A content (3.23 µg/100 g). There is limited literature regarding common bean characterization for vitamin A contents. To our knowledge, there is only one report regarding vitamin A content determination in common beans. Köse et al. [26] aimed to investigate the protein, mineral, and vitamin contents in locally grown common bean genotypes from Türkiye and found a good range of variations for various vitamins. They found that retinol (vitamin A) contents of the Pinarli genotype were significantly higher (4.15 μg 100 g^−1^) than the Yenice genotype (2.0 μg 100 g^−1^) in their study. Based on the average vitamin A content, our study reported lower vitamin A contents compared to the report by Köse et al. [26]. The differences in vitamin A contents could be due to the extraction protocol, specific germplasm used, or the locations at which they were grown. In a broader context, research conducted on other leguminous crops also revealed considerable variation in vitamin A content. For instance, a study in Nigeria reported that cowpeas contained vitamin A levels ranging from 7.46 to 37.42 µg/100 g [32], highlighting their potential as a dietary source of vitamin A. Additionally, a study by Arslan [33] documented retinol levels (a form of vitamin A) ranging from 25.6 to 44.1 µg/kg, alongside β-carotene concentrations of 240.8 to 410.1 µg/kg across different genotypes. These findings suggest that both crop type and genotype play a crucial role in vitamin A content variation.

In a broader context, previous work on other leguminous crops further demonstrated significant variation in vitamin A content. For example, a study in Nigeria revealed that cowpeas contained vitamin A levels ranging from 7.46 to 37.42 µg/100 g [32], emphasizing their potential role as a dietary source of vitamin A. Furthermore, Arslan [33] reported that retinol (vitamin A) ranged from 25.6 to 44.1 µg/kg in various genotypes. Results indicate that both crop type and genotype significantly contributed to the observed variation in vitamin A content. The variability regarding vitamin A content among Turkish common bean germplasm, as well as other legume crops, indicates the need for genotype selection, along with environmental factors and differences in methodology to determine vitamins. The germplasm examined was obtained from 19 different provinces of Türkiye, making it crucial to identify which regions hold vitamin A-enriched landraces. The analysis revealed that landraces from Balıkesir exhibited high vitamin A content, whereas accessions from Bilecik demonstrated comparatively low levels of vitamin A content (Figure 1). As shown in Table 2, majority of the landraces from Balıkesir exhibited vitamin A concentrations surpassing 3 µg/100 g of dry seed across both sites and under all five examined conditions. This study selected vitamin A-rich stable common bean accessions using parametric approaches. Shukla [34] proposed that genotypes with minimal σ^2^_i_ values are more stable. In this investigation, we also assessed ten common bean accessions that demonstrated exceptional vitamin A performance in five different settings (Table 4). These accessions have the potential to be beneficial for the breeding of common bean in terms of vitamin A.

In the context of marker–trait association for vitamin A content in Turkish common bean germplasm, a single marker (3367588) located on the Pv06 chromosome of the common bean genome showed a substantial association with vitamin A levels. Santos and Simon [35] performed QTL analysis that revealed clustered loci for the accumulation of major pro-vitamin A carotenes and lycopene in carrot roots. Vigun06g056300 present on the sixth chromosome of *Vigna unguiculate*, was found to be a putative candidate gene for the investigated marker. This gene encodes for the UDP-Glycosyltransferase protein superfamily. This protein family is involved in the transfer of glycosyl residues from activated nucleotide sugars to acceptor molecules (aglycones), thus regulating properties of the acceptors such as their bioactivity, solubility, and transport within the cell and throughout the organism [36]. To the best of our knowledge, this is the very first report claiming marker–trait association for vitamin A contents in legumes, especially in common beans. Identified markers through this study will be helpful in the marker-assisted breeding of common bean germplasm for vitamin A contents.

## 4. Materials and Methods

### 4.1. Plant Material

During this study, common bean germplasm consisting of 177 landraces and 6 commercial cultivars (Akman, Göynük, Karacaşehir, Önceler, Göksun, and Akdağ) were used as the plant materials. Each genotype was assigned a unique identifier (G1 to G183) for reference in figures and clustering analyses, corresponding to the specific accession numbers used in the genetic evaluation (Supplementary Table S1). This plant material was collected from 19 provinces in Türkiye, primarily from significant common bean cultivation areas, and preserved at Bolu Abant Izzet Baysal University (BAIBU). These commercial cultivars have been established as standard references in a previous study [37,38]. This plant material was previously studied, and has strong genetic diversity. Detailed information about the studied germplasm is given in Supplementary Table S1.

### 4.2. Field Experimentation

Seeds were sown by hand in elementary plots, each consisting of 2-m-long rows, with a 50 cm inter- and 10 cm intra-row spacing in an augmented design in BAIBU on 24, 27, and 17 April of 2016, 2017, and 2018, respectively. We followed the same sowing strategy for the second location, i.e., Cumhuriyet University (coordinates: 9.7087° N, 37.0203° E, altitude; 1293 m above mean sea level) in the Sivas province of Turkey on 15 and 12 April of 2017 and 2018, respectively. The soil of the experimental area of BAIBU has a loamy texture, a slightly alkaline character (pH 7.59), and low organic matter contents (1.80–1.86%). The experimental area of Sivas contained mainly silt (48.3%) and clay (37.1%), and a low content of organic matter (1.7%) with a pH of 7.28. Climatic conditions of both locations during the whole study period are presented in Table S2. The three experimental years in Bolu (2016, 2017, and 2018) and two in Sivas (2017, 2018), were considered as five environments for analytical purposes, as this is a common practice in agricultural experimentations [38]. After thinning, a total of 10 plants in each row for each accession were maintained for phenotypic characterization. Di-ammonium phosphate (DAP) and ammonium sulfate were used as a source of fertilizer. Four irrigations and three hoeings were performed during each growing season at both locations. Seeds were harvested at 90% pod maturity and were stored at optimum temperature (21–27 °C).

### 4.3. Seed Vitamin A Contents Profiling

Vitamin A contents in the seeds of common bean germplasm were investigated according to the methodology described by Akande et al. [16]. A total of 5 g seeds from each accession were taken and their grinding was performed. One gram of ground powder from each accession was placed in test tubes. A total of 5 mL of propanol (98%) was added into the test tube and incubated for 10 min to achieve the proper extract. Filtration of these samples was performed, and the resulting filtration was transferred into a cuvette. A UV-visible spectrophotometer was used for absorbance read at 325 nm by following the methodology described by A.O.A.C (1990). The absorbance was taken proportional to the vitamin A content in the sample. A single measurement was made.

### 4.4. Molecular Analysis

The DNA isolation was performed according to the CTAB protocol [39], and a specific protocol suggested by Diversity Arrays Technology (available at https://ordering.diversityarrays.com/files/DArT_DNA_isolation.pdf). The high-grade DNA was further diluted to achieve a final concentration of 50 ng μL^−1^. The diluted DNA (50 ng/ ng μL^–1^) was processed using Diversity Arrays Technology for GBS analysis. Detailed information about GBS analysis for the studied germplasm can be obtained from our published study [40].

### 4.5. Statistical Analysis

#### 4.5.1. Phenotypic Data Analysis

An online software developed by Rathore et al. [41] was used for statistical inferences. The analysis of variance (ANOVA) and genotypes by environment interaction was calculated according to the methodology described in our previous studies [21,25]. The analyses were conducted based on adjusted means. Statistics like mean, range, standard error, frequency distribution, and principal component analysis (PCA) were investigated through XLSTAT version 2021.3.1 (www.xlstat.com). To find the most stable accessions for vitamin A contents, an online program called “STABILITYSOFT” was utilized [42]. Most stable accessions were selected based on some parametric methods such as the Wricke’s equivalence stability index (W_i_^2^) [43], Shukla’s stability variance (σ^2^_i_) [34], and deviations from the regression (s^2^d_i_) [44]. The dendrogram was constructed through R statistical software version 3.4.1.

#### 4.5.2. Marker–Trait Association Analysis for Vitamin A Contents

Marker–trait association (MTA) analysis was carried out using a mixed linear model (MLM) approach (Q + K) following the same method as in our previous studies [40,45]. During the MTA investigation, we used the kinship (K) matrix as described by Bradbury et al. [46] and employed the TASSEL 5.0.5 software (https://tassel.bitbucket.io). The population structure of the germplasm under study was previously analyzed in our earlier research [40], where the Q matrix for each sample was determined based on the reported structural analysis. Both Bonferroni and false discovery rate (FDR) thresholds were applied. DArTseq markers meeting the significance criteria of FDR and Bonferroni *p* = 0.01 were identified as strongly linked with Mn content. The Manhattan plot in R 3.4.1 statistical software (http://www.r-project.org/) was employed to visualize statistically significant markers for Mn content, utilizing the qqman R package [47].

## 5. Conclusions

The present investigation revealed significant phenotypic diversity of vitamin A contents in Turkish common bean germplasm. Civril-Bolu and Bilecik-6 were found to have phenotypic diverse landraces for vitamin A contents, and it is suggested to use these landraces as parent candidates for vitamin A breeding of common beans. Landraces belonging to Balıkesir province were found to be rich in vitamin A contents, and should be considered a potential genetic resource for common bean biofortification. Marker–trait association analysis revealed 3367588 as a highly significant marker located on the sixth chromosome for vitamin A contents.

## Figures and Tables

**Table 1:** Analysis of variance (ANOVA) for vitamin A content in common bean genetic sources across different environments.

Source	Df	Sum Sq	Mean Sq	F Value	Pr (>F)
ENV	4	0.310747	0.077687	23.15593	2.95 × 10^−18^
REP (ENV)	5	0.067811	0.013562	4.042485	0.001236
GEN	182	155.7559	0.8465	252.3146	0
GEN:ENV	736	7.026215	0.009546	2.845504	1.53 × 10^−50^
Residuals	920	3.086542	0.003355		

ENV: environment, GEN: genotype, Df: degrees of freedom, Sum Sq: sum of squares, Mean Sq: mean of squares.

**Table 2:** Maximum, minimum, mean and standard deviation values of vitamin A content (µg/100 g) of common bean genotypes.

Years	Minimum	Maximum	Mean	Std. Deviation
Bolu17	1.670	3.220	2.395	0.293
Sivas17	1.660	3.260	2.395	0.310
Bolu18	1.660	3.350	2.412	0.284
Sivas18	1.630	3.240	2.404	0.298
Sivas21	1.650	3.230	2.397	0.294
Mean	1.670	3.230	2.400	0.292

**Table 3:** Principal component analysis results of common bean genotypes.

	PCA1	PCA2	PCA3	PCA4	PCA5
BOLU 2017	0.993	−0.061	−0.045	−0.072	−0.047
SİVAS 2017	0.992	−0.050	−0.058	0.096	−0.002
BOLU 2018	0.996	−0.046	0.011	−0.037	0.072
SİVAS 2018	0.987	0.158	−0.031	−0.008	0.002
SİVAS 2021	0.992	−0.001	0.123	0.020	−0.024
Eigenvalue	4.921	0.033	0.021	0.016	0.008
Variability (%)	98.419	0.668	0.429	0.323	0.160
Cumulative (%)	98.419	99.087	99.517	99.840	100.000

**Table 4:** Stability assessment of common bean landraces for Vitamin A content across five environments and two locations.

Genotype	Vitamin A Content	W_i_^2^	σ^2^_i_	s^2^d_i_
Niğde-Dermasyon	2.46	0.00013	1.08 × 10^−05^	9.53 × 10^−06^
Balıkesir-3	2.92	0.000228	3.05 × 10^−05^	2.78 × 10^−05^
Önceler	2.74	0.00046	7.74 × 10^−05^	4 × 10^−05^
Muş-34	2.3	0.000584	0.000103	4.75 × 10^−05^
Akdağ	2.54	0.000609	0.000108	5.61 × 10^−05^
Bitlis-5	2.51	0.00067	0.00012	9.57 × 10^−05^
Bilecik-6	1.67	0.000677	0.000121	4.46 × 10^−05^
Akman	2.98	0.000829	0.000152	3.64 × 10^−05^
Göksun	2.76	0.000829	0.000152	3.64 × 10^−05^
Göynük	2.46	0.00085	0.000156	7.21 × 10^−06^

W_i_^2^: Wricke’s ecovalence; σ^2^_i_: Shukla’s stability variance; s^2^d_i_: deviation from regression.

**Table 5:** Statistically significant related DArTseq markers for some vitamin contents in Turkish common bean gene sources.

Trait	Marker	Chromosome	Position	*p*-Value	MarkerR2
**Vitamin A**	3367588	6	16519249	3.57 × 10^−04^	0.07977

## Data Availability

All data needed to conduct this study is provided within the manuscript.

## Supplementary Materials

The following supporting information can be downloaded at: https://www.mdpi.com/article/10.3390/plants14050776/s1, Table S1: Passport data of 183 Turkish common bean accessions; Table S2: Vitamin A content (µg/100g) in the seeds of Turkish common bean germplasm.
