# Supplementary material for: Identification of Phenotypic Diversity and DArTseq Loci Associated with Vitamin A Contents in Turkish Common Bean Germplasm Through GWAS
Source: Plants (Basel). 2025 Mar 3;14(5):776. doi: 10.3390/plants14050776 (PMC11901511; doi:10.3390/plants14050776)
Supplement: Supplementary file 1 [file plants-14-00776-s001.zip › plants-3407528-supplementary.pdf]

Supplementary Data

Supplementary Table S1. Passport data of 183 Turkish common bean accessions.

G. No.	Names of Landraces	Collection Province	District	Village	Altitude (m)	Coordinates
G1	Bingol-1	Bingol	Genç	Selvi Beldesi	964	38° 34319/40° 18917
G2	Bingol-6	Bingol	Ilıcalar	Merkez	1161	38° 58893/40° 40699
G3	Bingol-7	Bingol	Merkez	Alatepe	1154	39° 03502/40° 45401
G4	Bingol-11	Bingol	Merkez	Çobantaşı	1542	39° 04033/40° 48557
G5	Bingol-16	Bingol	Adaklı	Gökçeli	1335	39° 12738/40° 25142
G6	Bingol-18	Bingol	Kiğı	Güneyağıl	1489	39° 17427/40° 20136
G7	Bingol-25	Bingol	Solhan	Kavaklıdere	1176	38° 55287/40° 56822
G8	Bingol-33	Bingol	Yedisu	Şen Mezrası	-	-
G9	Bingol-36	Bingol	Yedisu	Muz	-	-
G10	Bingol-44	Bingol	Yedisu	Kürdan	-	-
G11	Bingol-45	Bingol	Yedisu	Kürdan	-	-
G12	Bingol-52	Bingol	Yedisu	Eski Balta	-	-
G13	Bingol-53	Bingol	Yedisu	Eski Balta	-	-
G14	Bingol-58	Bingol	Yedisu	Kara Polat	-	-
G15	Bingol-60	Bingol	Yedisu	Döşengi	-	-
G16	Bingol-61	Bingol	Yedisu	Kara Polat	-	-
G17	Bingol-63	Bingol	Yedisu	Güzgülü	-	-
G18	Bingol-65	Bingol	Karlıova	Üçevler	-	-
G19	Hakkari-7	Hakkari	Merkez	Otluca	2054	37° 36246/43° 42370
G20	Hakkari-11	Hakkari	Merkez	Üzümcü	2097	37° 36332/43° 42526
G21	Hakkari-12	Hakkari	Merkez	Üzümcü	2097	37° 36332/43° 42526
G22	Hakkari-13	Hakkari	Merkez	Ağaçdibi	2097	37° 29370/43° 38184
G23	Hakkari-16	Hakkari	Merkez	Çimenli	1137	37° 29096/43° 37693
G24	Hakkari-20	Hakkari	Merkez	Üzümcü	1135	37° 29773/43° 34389
G25	Hakkari-23	Hakkari	Merkez	Taşbaşı	970	37° 23929/43° 29723
G26	Hakkari-28	Hakkari	Çukurca	Narlı	875	37° 16013/43° 35195

G27	Hakkari-31	Hakkari	Merkez	Bay	1832	37° 32687/43° 43333
G28	Hakkari-37	Hakkari	Merkez	Merzan	1993	37° 34095/43° 42308
G29	Hakkari-38	Hakkari	Merkez	Merzan	1993	37° 34095/43° 42308
G30	Hakkari-39	Hakkari	Merkez	Merzan	1993	37° 34095/43° 42308
G31	Hakkari-43	Hakkari	Merkez	Durankaya	1764	37° 33418/43° 37329
G32	Hakkari-44	Hakkari	Merkez	Durankaya	1764	37° 33418/43° 37329
G33	Hakkari-51	Hakkari	Merkez	Rezan	1601	37° 42104/43° 56276
G34	Hakkari-55	Hakkari	Yüksekova	Bağışlı	1811	37° 43279/44° 02206
G35	Hakkari-63	Hakkari	Yüksekova	Su Üstü	1955	37° 35208/43° 04488
G36	Hakkari-65	Hakkari	Yüksekova	Büyük Çiftlik	1955	37° 35208/43° 04488
G37	Hakkari-69	Hakkari	Yüksekova	Merkez	1915	37° 32928/44° 08427
G38	Hakkari-71	Hakkari	Şemdinli	Güzelkonak	1724	37° 25223/44° 29056
G39	Hakkari-76	Hakkari	Merkez	Üzümcü	1135	37° 29773/43° 34389
G40	Tokat-83	Tokat	-	-	-	-
G41	Maras-92	Kahramanmaraş	-	-	-	-
G42	Bitlis-5	Bitlis	Hizan	Merkez	1629	38° 13424/42° 21614
G43	Bitlis-14	Bitlis	Hizan	Akbıyık	1522	38° 11967/42° 20644
G44	Bitlis-16	Bitlis	Hizan	Yemişli	1638	38° 12806/42° 21679
G45	Bitlis-22	Bitlis	Hizan	Bahçelievler	1521	38° 12806/42° 21679
G46	Bitlis-25	Bitlis	Hizan	Kalkanlı	2004	38° 07704/42° 37670
G47	Bitlis-35	Bitlis	Hizan	Soğuksu	1365	38° 06783/42° 33292
G48	Bitlis-40	Bitlis	Hizan	Gayda	1271	38° 10051/42° 22985
G49	Bitlis-46	Bitlis	Tatvan	Yolalan	1645	38° 16080/42° 18559
G50	Bitlis-48	Bitlis	Merkez	Çınarbaşı	1710	38° 15861/42° 17972
G51	Bitlis-53	Bitlis	Merkez	Kuşlu	1615	38° 19739/42° 14841
G52	Bitlis-66	Bitlis	Mutki	Yumrumeşe	1459	38° 26765/41° 51660
G53	Bitlis-69	Bitlis	Mutki	Kavakbaşı	1303	38° 28884/41° 48924
G54	Bitlis-76	Bitlis	Mutki	Çiftlikyol	1259	38° 30098/41° 46302

G55	Bitlis-79	Bitlis	Mutki	Eller	1423	38° 28878/41° 43845
G56	Bitlis-81	Bitlis	Güroymak	Yazlıkonak	1810	38° 30257/42° 07150
G57	Bitlis-90	Bitlis	Güroymak	Aşağıkolbaşı	1655	38° 32695/42° 06804
G58	Bitlis-94	Bitlis	Güroymak	Arpacık	1700	38° 30930/42° 05787
G59	Bitlis-97	Bitlis	Güroymak	Kuştaşı	2002	38° 29645/42° 04575
G60	Bitlis-103	Bitlis	Tatvan	Taşdemir	1828	38° 27451/42° 23777
G61	Bitlis-105	Bitlis	Tatvan	Çamaltı	1728	38° 27483/42° 26602
G62	Bitlis-111	Bitlis	Tatvan	Reşadiye	1689	38° 29404/42° 32232
G63	Bitlis-114	Bitlis	Merkez	Çınarbaşı	1459	38° 26765/42° 51660
G64	Bitlis-115	Bitlis	Mutki	Yumrumeşe	2002	38° 29645/42° 04575
G65	Bitlis-117	Bitlis	Merkez	Kuşlu	1615	38° 19739/42° 14841
G66	Bitlis-118	Bitlis	Tatvan	Kırkbulak	1752	38° 24726/42° 16166
G67	Bitlis-119	Bitlis	Hizan	Yemişli	1638	38° 12806/42° 21679
G68	Bitlis-120	Bitlis	Merkez	Yolalan	1543	38° 17889/42° 15891
G69	Bitlis-121	Bitlis	Mutki	Yumrumeşe	1459	38° 26765/41° 51660
G70	Bitlis-124	Bitlis	Güroymak	Yazlıkonak	1615	38° 19739/42° 14841
G71	Malatya-3	Malatya	Doğanşehi r	Erkenek Bel.	1388	37° 55785/37° 56501
G72	Malatya-13	Malatya	Doğanşehi r	Kurucaova Bel	1369	37° 59707/38° 01503
G73	Malatya-14	Malatya	Doğanşehi r	Savaklı	1364	38° 02576/37° 54593
G74	Malatya-18	Malatya	Doğanşehi r	Elmalı	1410	38° 03339/37° 44688
G75	Malatya-25	Malatya	Doğanşehi r	Çığlık	1235	38° 06477/37° 55440
G76	Malatya-28	Malatya	Doğanşehi r	Güroba	1459	38° 05052/37° 57494
G77	Malatya-32	Malatya	Doğanşehi r	Çömlekoba	1370	38° 05372/37° 56691
G78	Malatya-33	Malatya	Doğanşehi r	Polat Bel.	1270	38° 09447/37° 51215
G79	Malatya-45	Malatya	Akçadağ	Ören	1158	38° 14905/37° 55605
G80	Malatya-50	Malatya	Hekimhan	Çayevleri Mah.	1457	38° 48854/37° 54964
G81	Malatya-51	Malatya	Yeşilyurt	Aşağıköy	1456	38° 09010/38° 18332

G82	Malatya-52	Malatya	Doğanşehi r	Merkez	1280	38° 06477/37° 55440
G83	Malatya-59	Malatya	Doğanşehi r	Kurucaova	1369	37° 59707/38° 01503
G84	Malatya-71	Malatya	Doğanşehi r	Güroba	1465	38° 05052/37° 57494
G85	Tunceli-1	Tunceli	Mazgirt	Merkez	1122	39° 00014/39° 34766
G86	Tunceli-5	Tunceli	Ovacık	Yeşilova	1289	39° 20037/39° 05286
G87	Tunceli-11	Tunceli	Pertek	Beydamı	-	-
G88	Van-1	Van	Gürpınar	Merkez	1748	38° 19126/43° 22555
G89	Van-11	Van	Çatak	Elmacı	1807	38° 04867/43° 04475
G90	Van-13	Van	Çatak	Bilgi	1702	38° 05736/43° 15575
G91	Van-17	Van	Çatak	Bilgi	1702	38° 05736/43° 15575
G92	Van-19	Van	Çatak	Alacayar	1629	38° 01890/43° 08884
G93	Van-25	Van	Çatak	Merkez	1502	38° 00451/43° 03619
G94	Van-27	Van	Çatak	Merkez	1783	38° 00721/43° 04473
G95	Van-33	Van	Başkale	Çaldıran	2005	37° 47409/44° 07448
G96	Van-36	Van	Başkale	Belliyurt	1876	37° 49064/44° 06905
G97	Van-42	Van	Erciş	Merkez	1704	39° 01746/43° 21668
G98	Van-47	Van	Erciş	Merkez	1689	39° 00036/43° 21362
G99	Van-51	Van	Başkale	Barış	2244	38° 01147/43° 39146
G100	Van-59	Van	Çatak	Elmacı	1807	38º 04867 / 43º 04475
G101	Van-64	Van	Bahçesara y	Ünlüce	1702	38° 31128/42° 19587
G102	Van-65	Van	Bahçesara y	Ünlüce	1702	38° 31128/42° 19587
G103	Van-68	Van	Bahçesara y	Elmayaka	1705	38° 30546/42° 19126
G104	Elazig-2	Elazig	Palu	Seydilli	877	38° 41578/39° 53162
G105	Elazig-7	Elazig	Palu	Gömeçbağlar	956	38° 37887/39° 51625
G106	Elazig-9	Elazig	Palu	Keklikdere	870	38° 36885/39° 49865
G107	Elazig-10	Elazig	Palu	Baltaşı	919	38° 35361/39° 47344
G108	Elazig-14	Elazig	Maden	Gezin	919	38° 35361/39° 47344
G109	Elazig-16	Elazig	Maden	Kızıltepe	1291	38° 28865/39° 31155

G110	Elazig-25	Elazig	Maden	Yıldızhan	1313	38° 21174/39° 22660
G111	Elazig-27	Elazig	Sivrice	Başkaynak	1390	38° 22855/39° 22217
G112	Elazig-29	Elazig	Sivrice	Elmasuyu	1364	38° 24728/39° 23341
G113	Elazig-30	Elazig	Maden	Gezin	1350	38° 30760/39° 33182
G114	Elazig-34	Elazig	Maden	Yeşilova	1503	38° 32905/39° 33695
G115	Elazig-36	Elazig	Maden	Küçükova	1410	38° 32551/39° 32526
G116	Elazig-39	Elazig	Maden	Gezin	1350	38° 30760/39° 33182
G117	Mus-1	Mus	Malazgirt	Gülkuru	1607	39° 05869/42° 38738
G118	Mus-2	Mus	Bulanık	Güllüova	1550	39° 03619/42° 19105
G119	Mus-7	Mus	Bulanık	Güllüova	1550	39° 03619/42° 19105
G120	Mus-10	Mus	Bulanık	Balotu	1489	39° 06752/42° 08046
G121	Mus-15	Mus	Bulanık	Değirmensuy u	1514	39° 10268/42° 05099
G122	Mus-18	Mus	Korkut	Sazlıkbaşı	1293	39° 40424/41° 58975
G123	Mus-22	Mus	Hasköy	Merkez	1315	38° 38175/41° 46056
G124	Mus-27	Mus	Hasköy	Azıklı	1369	38° 38595/41° 44016
G125	Mus-28	Mus	Hasköy	Kültür	1278	38° 40889/41° 41773
G126	Mus-34	Mus	Merkez	Akpınar	1400	39° 10591/41° 30486
G127	Mus-39	Mus	Varto	Tepeköy	1280	39° 05383/41° 30168
G128	Mus-41	Mus	Varto	Tepeköy	1280	39° 05383/41° 30168
G129	Mus-42	Mus	Varto	Özenç	1468	39° 06895/41° 30281
G130	Mus-43	Mus	Varto	Taşçı	1577	39° 12636/41° 23917
G131	Mus-46	Mus	Bulanık	Güllüova	1550	39° 03619/42° 19105
G132	Mus-48	Mus	Bulanık	Güllüova	1550	39° 03619/42° 19105
G133	Mus-49	Mus	Bulanık	Güllüova	1550	39° 03619/42° 19105
G134	Mus-50	Mus	Bulanık	Balotu	1489	39° 06752/42° 08046
G135	Mus-51	Mus	Bulanık	Adıvar	1463	38° 13447/42° 10513
G136	Mus-52	Mus	Hasköy	Merkez	1350	38° 13447/42° 10513

G137	Mus-53	Mus	Hasköy	Azıklı	1369	38° 38595/41° 44016
G138	Sivas-3	Sivas	Suşehri	Arpacı	1050	40° 957/38° 539
G139	Sivas-4	Sivas	Suşehri	Günlüce	1050	40° 957/38° 539
G140	Sivas-7	Sivas	Suşehri	Akşar	1050	40° 957/38° 539
G141	Sivas-12	Sivas	Hafik	Yakaboyu	1350	39° 510/37° 230
G142	Sivas-13	Sivas	Kangal	Akpınar	1540	39° 130/37° 240
G143	Sivas-16	Sivas	Divriği	Arıkbaşı	1250	39° 240/38° 70
G144	Sivas-17	Sivas	İmranlı	Başlıca	1650	39° 5248/38° 758
G145	Sivas-18	Sivas	İmranlı	Gökdere	1650	39° 5248/38° 758
G146	Sivas44	-	-	-	-	-
G147	Sivas62	-	-	-	-	-
G148	Sivas68	-	-	-	-	-
G149	Sivas69	Sivas	-	-	-	-
G150	Sivas-70	Sivas	-	-	-	-
G151	Bilecik-1	Bilecik	Pazaryeri	Dereköy	786	39° 59′38″/29° 54′41″
G152	Bilecik-2	Bilecik	Pazaryeri	Günyurdu	805	40° 0′5.9″/29° 54′9″
G153	Bilecik-6	Bilecik	Pazaryeri	Dereköy	876	39° 59′38″/29° 54′41″
G154	Bilecik-7	Bilecik	Pazaryeri	Dereköy	876	39° 59′38″/29° 54′41″
G155	Bilecik-10	Bilecik	Pazaryeri	Dereköy	876	39° 59′38″/29° 54′41″
G156	Balikesir-3	Balikesir	Manyas	Salur Mah.	29	40° 05′51″/27° 56′11″
G157	Balikesir-4	Balikesir	Manyas	Akçaova Mah.	30	40° 07′16”/27° 51′18″
G158	Balikesir-5	Balikesir	İvrindi	Ayaklı Köyü	404	39.516°/27.364°
G159	Balikesir-6	Balikesir	İvrindi	Ayaklı Köyü	403	39.516°/27.364°
G160	Balikesir-17	Balikesir	Sındırgı	Kürendere	1051	39.313°/28.571°
G161	Balikesir-18	Balikesir	Sındırgı	Kürendere	1051	39.313°/28.571°
G162	Balikesir-19	Balikesir	Sındırgı	Kürendere	1051	39.313°/28.571°
G163	Balikesir-20	Balikesir	Sındırgı	Kürendere	1051	39.313°/28.571°
G164	Duzce-1	Duzce	Merkez	Derdin	859	40.711°/31.228°
G165	Duzce-9	Duzce	Merkez	Darıca Mah.	163	40° 49′18″/31° 10′26″
G166	Yalova-13	Yalova	Çiftlikköy	Kabaklı	125	40° 39′30″/29° 24′36″
G167	Yalova-20	Yalova	Çınarcık	Ortaburun	689	40° 37′04″/29° 09′00″
G168	Yalova-21	Yalova	Çınarcık	Ortaburun	688	40° 37′04″/29° 09′00″
G169	Erzincan-1	Erzincan	Refahiye	Merkez	1589	39° 544/38° 467
G170	Erzincan-3	Erzincan	Kemah	Gökkaya	1130	39° 3610/39° 28
G171	Erzincan-4	Erzincan	Kemaliye	Merkez	950	39° 1539/38° 2948
G172	Erzincan-5	Erzincan	Kemaliye	Akçalı	950	39° 1539/38° 2948

G173	Bursa-1	Bursa	Yenişehir	Fethiye	335	40.289°/29.445°
G174	Bursa-22	Bursa	Kestel	Aksu	360	40.169°/29.317°
G175	Nigde-Dermasyon	Nigde	-	-	-	-
G176	Nigde-Derinkiyu	Nigde	-	-	-	-
G177	Civril-Bolu	Bolu	Merkez	Doğancı Mah.	842	40° 40′45″/31° 33′30″
G178	Akman ×					
G179	Goynuk ×					
G180	Karacasehir ×					
G181	Onceler×					
G182	Goksun×					
G183	Akdag×					

× = Cultivars

Supplementary Table S2. Vitamin A content (µg/100g) in the seeds of Turkish common bean germplasm.

Genotypes	Bolu17	Sivas 17	Bolu18	Sivas18	Sivas21	Mean of Environments
**Bingöl-1**	2.17	2.18	2.2	2.23	2.18	2.19
**Bingöl-6**	2.22	2.19	2.28	2.21	2.26	2.23
**Bingöl-7**	2.36	2.42	2.35	2.31	2.36	2.36
**Bingöl-11**	2.31	2.36	2.33	2.36	2.33	2.34
**Bingöl-16**	2.23	2.22	2.26	2.28	2.23	2.24
**Bingöl-18**	2.34	2.33	2.32	2.42	2.32	2.35
**Bingöl-25**	2.42	2.38	2.43	2.4	2.4	2.41
**Bingöl-33**	2.28	2.25	2.2	2.19	2.3	2.24
**Bingöl-36**	2.28	2.29	2.31	2.26	2.31	2.29
**Bingöl-44**	2.58	2.62	2.54	2.51	2.56	2.56
**Bingöl-45**	2.36	2.37	2.4	2.36	2.4	2.38
**Bingöl-52**	2.51	2.56	2.5	2.6	2.51	2.54
**Bingöl-53**	2.37	2.35	2.41	2.46	2.36	2.39
**Bingöl-58**	2.47	2.56	2.26	2.49	2.46	2.45
**Bingöl-60**	2.12	2.03	2.19	2.06	2.09	2.1
**Bingöl-61**	2.55	2.65	2.34	2.53	2.55	2.52
**Bingöl-63**	2.65	2.66	2.59	2.55	2.65	2.62

**Bingöl-65**	2.51	2.52	2.65	2.57	2.54	2.56
**Hakkari-7**	2.51	2.53	2.54	2.58	2.52	2.54
**Hakkari-11**	2.42	2.42	2.41	2.5	2.42	2.43
**Hakkari-12**	2.52	2.52	2.59	2.56	2.51	2.54
**Hakkari-13**	2.49	2.5	2.47	2.49	2.5	2.49
**Hakkari-16**	2.27	2.23	2.3	2.22	2.26	2.26
**Hakkari-20**	2.48	2.55	2.47	2.43	2.49	2.48
**Hakkari-23**	2.26	2.24	2.27	2.31	2.26	2.27
**Hakkari-28**	2.31	2.29	2.29	2.32	2.31	2.3
**Hakkari-31**	2.19	2.17	2.2	2.27	2.18	2.2
**Hakkari-37**	2.27	2.33	2.23	2.36	2.26	2.29
**Hakkari-38**	2.34	2.36	2.33	2.31	2.32	2.33
**Hakkari-39**	2.68	2.81	2.49	2.63	2.7	2.66
**Hakkari-43**	2.19	2.13	2.3	2.09	2.21	2.18
**Hakkari-44**	1.93	1.79	2.24	2.01	1.94	1.98
**Hakkari-51**	2.67	2.67	2.69	2.73	2.68	2.69
**Hakkari-55**	2.31	2.26	2.4	2.39	2.32	2.34
**Hakkari-63**	2.37	2.42	2.36	2.39	2.38	2.38
**Hakkari-65**	2.35	2.33	2.4	2.36	2.38	2.36
**Hakkari-69**	2.18	2.22	2.23	2.14	2.17	2.19
**Hakkari-71**	2.4	2.43	2.41	2.39	2.41	2.41
**Hakkari-76**	2.63	2.69	2.61	2.65	2.6	2.64
**Tokat-83**	2.45	2.4	2.55	2.53	2.46	2.48
**Kahramanmaraş-92**	2.67	2.69	2.66	2.7	2.66	2.67
**Bitlis-5**	2.49	2.51	2.51	2.53	2.5	2.51
**Bitlis-14**	2.37	2.35	2.32	2.32	2.37	2.34
**Bitlis-16**	2.4	2.33	2.49	2.29	2.42	2.39
**Bitlis-22**	2.41	2.41	2.47	2.43	2.43	2.43

**Bitlis-25**	2.54	2.67	2.38	2.62	2.53	2.55
**Bitlis-35**	2.39	2.33	2.55	2.47	2.38	2.42
**Bitlis-40**	1.93	1.86	2.18	1.95	1.88	1.96
**Bitlis-46**	2.3	2.28	2.3	2.33	2.31	2.31
**Bitlis-48**	2.08	2.05	2.12	2.07	2.08	2.08
**Bitlis-53**	2.08	2.06	2.12	2.04	2.11	2.08
**Bitlis-66**	2.43	2.47	2.4	2.39	2.43	2.42
**Bitlis-69**	2.59	2.55	2.63	2.65	2.61	2.61
**Bitlis-71**	2.23	2.18	2.26	2.23	2.23	2.22
**Bitlis-79**	2.75	2.76	2.78	2.82	2.77	2.78
**Bitlis-81**	2.27	2.28	2.29	2.23	2.24	2.26
**Bitlis-90**	2.39	2.37	2.41	2.38	2.42	2.39
**Bitlis-94**	2.28	2.3	2.34	2.32	2.31	2.31
**Bitlis-97**	2.35	2.37	2.32	2.42	2.35	2.36
**Bitlis-103**	2.12	2.1	2.25	2.13	2.1	2.14
**Bitlis-105**	2.5	2.43	2.51	2.53	2.51	2.49
**Bitlis-111**	2.52	2.42	2.57	2.4	2.51	2.48
**Bitlis-114**	2.62	2.72	2.38	2.57	2.6	2.58
**Bitlis-115**	2.48	2.35	2.8	2.48	2.52	2.53
**Bitlis-117**	2.52	2.57	2.47	2.46	2.5	2.5
**Bitlis-118**	2.23	2.21	2.33	2.23	2.26	2.25
**Bitlis-119**	2.49	2.49	2.51	2.57	2.49	2.51
**Bitlis-120**	2.41	2.4	2.4	2.48	2.41	2.42
**Bitlis-121**	2.37	2.4	2.34	2.36	2.33	2.36
**Bitlis-124**	2.56	2.52	2.62	2.58	2.57	2.57
**Malatya-3**	2.11	2.04	2.15	2.08	2.12	2.1
**Malatya-13**	2.32	2.35	2.25	2.23	2.32	2.3
**Malatya-14**	2.09	2.08	2.25	2.16	2.13	2.14

**Malatya-18**	2.12	2.14	2.11	2.15	2.1	2.12
**Malatya-25**	2.51	2.49	2.54	2.59	2.53	2.53
**Malatya-28**	2.68	2.72	2.59	2.7	2.66	2.67
**Malatya-32**	2.1	2.04	2.27	2.15	2.11	2.13
**Malatya-33**	2.25	2.34	2.06	2.2	2.28	2.23
**Malatya-45**	2.04	2.03	2.18	1.99	2.04	2.06
**Malatya-50**	2.47	2.56	2.26	2.41	2.48	2.44
**Malatya-51**	2.13	1.99	2.41	2.18	2.13	2.17
**Malatya-52**	2.09	2.04	2.14	2.16	2.06	2.1
**Malatya-59**	2.28	2.29	2.33	2.36	2.26	2.3
**Malatya-71**	2.37	2.37	2.4	2.43	2.38	2.39
**Tunceli-1**	2.01	2.04	1.95	1.99	2	2
**Tunceli-5**	2.03	2.05	2.05	1.97	2.01	2.02
**Tunceli-11**	2.12	2.13	2.15	2.13	2.16	2.14
**Van-1**	2.61	2.58	2.7	2.68	2.62	2.64
**Van-11**	2.47	2.48	2.45	2.53	2.45	2.48
**Van-13**	2.13	2.08	2.22	2.21	2.12	2.15
**Van-17**	1.98	1.97	1.9	1.91	1.96	1.94
**Van-19**	1.99	2.03	2.01	2.01	1.99	2.01
**Van-25**	2.18	2.19	2.16	2.2	2.22	2.19
**Van-27**	1.96	1.95	2.1	1.91	1.96	1.97
**Van-33**	2.06	2.01	2.24	2.12	2.07	2.1
**Van-36**	2.26	2.34	2.09	2.34	2.26	2.26
**Van-42**	1.83	1.76	1.85	1.79	1.84	1.81
**Van-47**	2.32	2.31	2.33	2.25	2.32	2.31
**Van-51**	2.68	2.69	2.63	2.58	2.69	2.65
**Van-59**	1.97	1.98	2.04	2.06	1.98	2.01
**Van-64**	1.93	1.92	2.02	2.01	1.93	1.96

**Van-65**	2.12	2.13	2.13	2.22	2.1	2.14
**Van-68**	1.87	1.87	1.94	1.93	1.86	1.89
**Elazığ-2**	2.76	2.76	2.79	2.73	2.74	2.76
**Elazığ-7**	2.93	2.94	2.92	2.88	2.95	2.92
**Elazığ-9**	2.62	2.66	2.61	2.54	2.64	2.62
**Elazığ-10**	2.73	2.77	2.73	2.69	2.73	2.73
**Elazığ-14**	2.52	2.42	2.64	2.51	2.53	2.52
**Elazığ-16**	2.77	2.78	2.85	2.88	2.78	2.81
**Elazığ-25**	2.91	3.01	2.7	2.96	2.89	2.9
**Elazığ-27**	2.52	2.48	2.67	2.55	2.5	2.54
**Elazığ-29**	3.06	3.16	2.85	3.05	3.06	3.04
**Elazığ-30**	2.2	2.28	2.2	2.17	2.19	2.21
**Elazığ-34**	2.25	2.25	2.37	2.3	2.27	2.29
**Elazığ-36**	2.39	2.37	2.49	2.49	2.41	2.43
**Elazığ-39**	2.45	2.51	2.43	2.45	2.42	2.46
**Muş-1**	2.66	2.64	2.63	2.64	2.64	2.64
**Muş-2**	2.75	2.69	2.73	2.65	2.75	2.71
**Muş-7**	2.49	2.51	2.46	2.47	2.49	2.48
**Muş-10**	2.59	2.6	2.66	2.55	2.6	2.6
**Muş-15**	2.21	2.19	2.31	2.21	2.23	2.23
**Muş-18**	2.18	2.22	2.22	2.26	2.17	2.21
**Muş-22**	2.32	2.33	2.33	2.39	2.3	2.33
**Muş-27**	2.39	2.46	2.27	2.37	2.34	2.36
**Muş-28**	2.52	2.43	2.66	2.53	2.53	2.54
**Muş-34**	2.31	2.3	2.31	2.29	2.31	2.3
**Muş-39**	2.56	2.62	2.35	2.52	2.58	2.53
**Muş-41**	2.11	2.27	1.98	2.15	2.12	2.13
**Muş-42**	1.94	1.78	2.24	2.01	1.95	1.99

**Muş-43**	2.3	2.33	2.32	2.36	2.3	2.32
**Muş-46**	2.15	2.12	2.24	2.24	2.14	2.18
**Muş-48**	2.2	2.2	2.24	2.27	2.22	2.23
**Muş-49**	2.34	2.4	2.26	2.29	2.31	2.32
**Muş-50**	2.89	2.87	2.92	2.85	2.92	2.89
**Muş-51**	1.99	2.01	2.04	1.93	2	1.99
**Muş-52**	2.23	2.21	2.21	2.18	2.25	2.22
**Muş-53**	2.7	2.71	2.64	2.68	2.68	2.68
**Sivas-3**	2.74	2.68	2.84	2.81	2.75	2.76
**Sivas-4**	2.84	2.88	2.85	2.92	2.82	2.86
**Sivas-7**	2.69	2.69	2.68	2.71	2.68	2.69
**Sivas-12**	2.5	2.53	2.47	2.47	2.54	2.5
**Sivas-13**	2.42	2.41	2.53	2.37	2.4	2.42
**Sivas-16**	2.3	2.3	2.36	2.31	2.34	2.32
**Sivas-17**	2.82	2.9	2.66	2.89	2.83	2.82
**Sivas-18**	2.48	2.56	2.31	2.56	2.49	2.48
**Sivas-44**	1.93	1.78	2.09	1.84	1.89	1.9
**Sivas-62**	1.74	1.7	1.66	1.63	1.75	1.7
**Sivas-63**	2.45	2.43	2.5	2.44	2.46	2.45
**Sivas-69**	2.29	2.37	2.29	2.25	2.29	2.3
**Sivas-70**	2.41	2.43	2.43	2.43	2.45	2.43
**Bilecik-1**	2.08	2.05	2.06	2.08	2.05	2.06
**Bilecik-2**	1.74	1.72	1.75	1.81	1.75	1.75
**Bilecik-6**	1.67	1.66	1.7	1.68	1.65	1.67
**Bilecik-7**	1.9	1.92	1.9	1.93	1.9	1.91
**Bilecik-10**	2	2.01	1.94	1.9	2	1.97
**Balıkesir-3**	2.91	2.92	2.93	2.91	2.92	2.92
**Balıkesir-4**	3.07	3.13	3.05	3.15	3.07	3.1

**Balıkesir-5**	2.96	2.9	3.14	3.03	2.97	3
**Balıkesir-6**	3.13	3.13	3.18	3.24	3.11	3.16
**Balıkesir-17**	2.68	2.59	2.83	2.71	2.7	2.7
**Balıkesir-18**	3.15	3.26	2.93	3.13	3.14	3.12
**Balıkesir-19**	2.75	2.6	3.02	2.71	2.77	2.77
**Balıkesir-20**	2.98	3.01	2.92	2.9	2.96	2.96
**Düzce-1**	2.56	2.51	2.6	2.6	2.57	2.57
**Düzce-9**	2.3	2.29	2.26	2.31	2.28	2.29
**Yalova-13**	2.6	2.64	2.63	2.72	2.6	2.64
**Yalova-20**	2.18	2.19	2.24	2.21	2.16	2.2
**Yalova-21**	2.23	2.25	2.2	2.22	2.23	2.23
**Erzincan-1**	1.96	1.92	2.04	1.95	2	1.97
**Erzincan-3**	2.68	2.66	2.8	2.71	2.71	2.71
**Erzincan-4**	2.5	2.5	2.49	2.53	2.49	2.5
**Erzincan-5**	2.29	2.32	2.31	2.33	2.3	2.31
**Bursa-1**	2.73	2.68	2.73	2.78	2.74	2.73
**Bursa-22**	2.14	2.05	2.2	2.03	2.14	2.11
**Niğde-Dermasyon**	2.45	2.45	2.48	2.46	2.46	2.46
**Niğde-Derinkuyu**	2.72	2.86	2.53	2.68	2.72	2.7
**Civril-Bolu**	3.22	3.19	3.35	3.15	3.23	3.23
**Akman**	2.98	2.97	2.97	2.97	2.99	2.98
**Göynük**	2.45	2.45	2.5	2.47	2.45	2.46
**Karacaşehir**	2.79	2.82	2.79	2.81	2.79	2.8
**Önceler**	2.75	2.74	2.75	2.73	2.74	2.74
**Göksun**	2.76	2.75	2.75	2.75	2.77	2.76
**Akdağ**	2.54	2.52	2.56	2.56	2.53	2.54
